# Maternal Effects of Respiratory Syncytial Virus Infection during Pregnancy

**DOI:** 10.3201/eid2111.150497

**Published:** 2015-11

**Authors:** Sarahn M. Wheeler, Sarah Dotters-Katz, R. Phillip Heine, Chad A. Grotegut, Geeta K. Swamy

**Affiliations:** Duke University School of Medicine, Durham, North Carolina, USA

**Keywords:** respiratory syncytial virus, pregnancy, maternal, infection, viruses, RSV

## Abstract

Given the illness and deaths caused by respiratory syncytial virus (RSV) infection during the first year of life, preventing infant RSV infections through maternal vaccination is intriguing. However, little is known about the extent and maternal effects of RSV infection during pregnancy. We describe 3 cases of maternal RSV infection diagnosed at a US center during winter 2014. Case-patient 1 (26 years old, week 33 of gestation) received a diagnosis of RSV infection and required mechanical ventilation. Case-patient 2 (27 years old, week 34 of gestation) received a diagnosis of infection with influenza A(H1N1) virus and RSV and required mechanical ventilation. Case-patient 3 (21 years old, week 32 of gestation) received a diagnosis of group A streptococcus pharyngitis and RSV infection and was monitored as an outpatient. Clarifying the effects of maternal RSV infection could yield valuable insights into potential maternal and fetal benefits of an effective RSV vaccination program.

## Abstract 2

Clarifying these effects could show potential benefits of RSV vaccination of pregnant women.

Respiratory syncytial virus (RSV) is a major cause of serious, potentially fatal, respiratory infection in infants, but no preventive vaccine is available. In addition to ongoing research and development of an RSV vaccine for infants and children, the strategy of maternal vaccination, leading to passive transplancental transfer of anti-RSV antibodies, has been proposed. A recent animal study demonstrated passive transfer of RSV antibodies that controlled RSV pulmonary virus load in the offspring after maternal vaccination with a formalin-inactivated RSV vaccine (*1*). Although active research that examines an RSV vaccine in pregnancy is underway (*2**,**3*), the potential for maternal benefit that may be derived from such a vaccination program is unexplored.

Because of physiologic changes in cardiorespiratory function and obligatory immunologic alterations to allow for fetal tolerance, pregnant women are vulnerable to viral infections, such as influenza. In addition to maternal complications, including hospitalization, cardiorespiratory complications, and death, maternal influenza infection is associated with increased incidence of spontaneous abortion, fetal death, preterm birth, and low-birthweight infants (*4*). Consequently, the Centers for Disease Control and Prevention’s Advisory Committee on Immunization Practice recommends vaccination with inactivated influenza virus for all women who will be pregnant or postpartum during influenza season. However, little has been documented on the incidence and effects of maternal RSV infection on mother or infant. Only recently was RSV recognized as a key pathogen in respiratory infections afflicting elderly and immunocompromised adults; the estimated disease incidence was similar to that of nonpandemic influenza (*1*). It is likely that RSV is an important, yet unrecognized, pathogen in pregnancy. We describe 3 cases of antepartum RSV infection treated at a single tertiary care facility from January to March of 2014. These cases highlight the variability of clinical features, maternal and neonatal disease, and current lack of effective therapy for maternal RSV infection.

## Case-Patients

### Case-Patient 1

The first case occurred in a 26-year-old primagravida at week 33 of gestation who initially sought care at an urgent care facility for a 5-day history of malaise, cough, and wheezing. She reported recent exposure to a young child with an upper respiratory infection. Her medical history was notable for asthma, hypothyroidism, and gastresophageal reflux. Her social history was remarkable for smoking half of a pack of cigarettes per day and using marijuana daily. She had declined influenza vaccination during pregnancy.

The patient received a diagnosis of bronchitis and was treated with a β-agonist inhaler and amoxicillin. The next day she went to her local hospital when her symptoms worsened with new-onset fever. A chest radiograph showed no notable findings, and oxygen saturation was 94% on room air. With a diagnosis of worsening acute bronchitis, she was admitted to the hospital and administered ceftriaxone. Over the next few days, an oxygen requirement developed, and bilateral opacities were shown on chest x-ray, which evoked concern for possible acute respiratory distress syndrome. Azithromycin, vancomycin, and oseltamivir were empirically added to her treatment regimen, and imaging demonstrated no evidence of pulmonary embolism. Although she received broad-spectrum antibiotic drugs, her condition continued to deteriorate, and she required 100% fraction of inspired oxygen on a nonrebreather mask. She was therefore intubated and transferred to a tertiary care facility on hospital day (HD) 4.

On arrival at our institution, the patient was taken to the medical intensive care unit (ICU) where she remained intubated on assist control with 100% fraction of inspired oxygen, positive end–expiratory pressure of 16 cm H_2_O, and inspiratory pressure of 16 cm H_2_O. She continued to receive the previously prescribed antibiotic regimen, and a bronchoscopy was performed with collection of bronchial washings. The washings were analyzed by using the eSensor Respiratory Panel (GenMarkDx, Carlsbad, CA, USA), a multiplex, reverse transcription nucleic acid amplification test designed to detect influenza viruses A and B, RSV, and rhinovirus. PCR of respiratory specimens showed positive results for RSV. Additional tests for acid-fast bacilli, *Legionella* spp*.*, *Streptococcus pneumoniae*, *Pneumocystis* spp., and parainfluenza virus were all negative. All antibiotic drugs were discontinued at this time. Ribavarin was considered but was not administered because it is likely to be most effective when started early in the disease course, and its potential benefit in adults with RSV infection appears to be limited to immunocompromised adults (*5**–**8*).

The patient’s respiratory status improved with supportive therapy, and she was extubated on HD5. However, stridor and oxygen desaturation later developed, which required re-intubation. On HD6, a fever developed, and she was started on a course of vancomycin and piperacillin-tazobactam for presumed hospital-acquired pneumonia. Blood cultures were negative, and her respiratory status remained stable while she was intubated. Because her respiratory status did not improve enough to permit extubation, the decision was made to proceed with cesarean delivery to potentially accelerate her recovery by decreasing overall cardiorespiratory demand. On HD8, at 34 weeks and 1 day of gestation, the patient remained intubated and was transferred to the operating room, where she underwent an uncomplicated cesarean delivery of a male infant weighing 2,007 g and with 1- and 5-minute Apgar scores of 5 and 7, respectively. The infant required blow-by oxygen at birth and was admitted to the neonatal ICU. He was quickly weaned to room air. There was no clinical indication that the infant was infected with RSV, and therefore no virus testing was done. The infant was discharged to his home on day of life 8 without complication.

Two days after delivery, the patient’s respiratory status improved and she was extubated. She received oxygen by nasal cannula and was transferred to the medicine floor. Antibiotic drugs were discontinued, and she was given daily beclomethasone and albuterol, which substantially improved wheezing. She was discharged on HD14 with normal oxygen saturation levels on room air.

### Case-Patient 2

The second case-patient was a 27-year-old gravida 2, para 0, at week 34 and day 4 of gestation who sought treatment with worsening cough, congestion, fevers, and chills. Her pregnancy was complicated by fetal hydrocephalus consistent with aqueductal stenosis, which was diagnosed during the second trimester and was therefore unrelated to her pulmonary complications. Her fetus was noted to be in the breech presentation. Her prior obstetric history was notable for a spontaneous abortion at 8 weeks, and her medical history was notable for childhood asthma and smoking one half to 1 pack of cigarettes per day before pregnancy. She received an influenza vaccine during pregnancy.

Five days before seeking treatment at our institution, she was seen in a local emergency room for worsening cough and dyspnea; a rapid influenza test was negative, and she was treated with a 5-day course of prednisone and albuterol for exacerbation of asthma. Three days later, fever developed, and her cough was productive for green sputum; at that time she was given azithromycin. When she was admitted to our institution after no improvement in her illness, leukocyte count was 21 × 10^9^ cells/L (normal range 5.9–16.3 × 10^9^ cells/L) and chest radiograph showed a nonspecific opacity in the right middle lobe, which evoked concern for pneumonia. A nasopharyngeal aspirate was sent to be tested by viral respiratory PCR panel (eSensor; GenMarkDx). The panel was positive for influenza A(H1N1) virus and RSV. The patient was given ceftriaxone, vancomycin, azithromycin, and oseltamavir because of concern for secondary bacterial infection. Her oxygen saturation was stable (91%–94% on room air). On the afternoon of HD2, her membranes ruptured. Because of her pulmonary status, the decision was made to defer delivery because there were no signs of active labor. On HD3 at week 34 and day 5 of gestation, she reported contractions, and cervical dilation was noted. Given the fetal hydrocephalus (biparietal diameter 11.6 cm) and breech presentation, she underwent a classical cesarean delivery of a female infant weighing 3,640 g and with 1- and 5-minute Apgar scores of 3 and 7, respectively.

During the cesarean closure, the patient required intubation and conversion to general anesthesia owing to increased secretions, hypotension, and tachycardia. She remained intubated and was transported to the surgical ICU after delivery. She was extubated without difficulty within an hour of ICU admission and transferred to the postpartum unit 12 hours after delivery. While recovering in the postpartum unit, she had an episode of pulmonary edema that was successfully treated with furosemide. The remainder of her postpartum course was uncomplicated, and she completed a regimen of 10 days of azithromycin and 5 days of oseltamivir. She was discharged to her home on HD10 with percentages of oxygen saturation in the mid-90s on room air. The infant did well from a respiratory standpoint and was discharged on day of life 20 after an uncomplicated shunt placement due to hydrocephalus; to date, the infant is doing well.

### Case-Patient 3

The third case-patient was a 21-year-old primagravida who sought treatment at our outpatient clinic at week 32 of gestation with a 3-day history of sore throat, congestion, and fever. Her medical history was complicated by mild aortic coarctation and a cognitive delay. She had no history of asthma or smoking and had been vaccinated against influenza during pregnancy. At her initial visit, she was afebrile with stable vital signs and was therefore discharged to her home with empiric oseltamivir. A throat culture specimen was positive for group A streptococcus, and a nasopharyngeal aspirate (eSensor; GenMarkDx) was positive for RSV by viral PCR. The patient was treated with penicillin (500 mg, twice a day, for 10 days) for the group A streptococcus infection without any further complications. At 39 weeks of gestation, she underwent an uncomplicated primary cesarean delivery of a 2,855-g female infant with Apgar scores of 9 at 1 and 5 minutes. The infant was admitted to the newborn nursery and discharged home with the mother on postoperative day 4.

## Discussion

The 3 case-patients described highlight the underrecognized effects of maternal RSV infection on maternal and neonatal outcomes. It is well documented that despite nearly universal exposure to the virus by age 2, previous RSV infection does not confer long-term immunity; reinfection has even been documented within the same season (*9*). For most immunocompetent adults these reinfections are milder than the original infection and produce a cold-like illness that is self-limited. However, RSV infection–related complications and sequelae may be more serious in the setting of pregnancy. The patients in our study with the first and second cases required ICU level care and intubation, which suggests that maternal RSV infection can lead to clinically severe maternal disease. Although the infection in the third case-patient was self-limited, and she was treated as an outpatient, it indicates that maternal RSV may be a critical factor in mild respiratory illness that leads to physician visits, lost work time, and possible inappropriate administration of antibiotic drugs.

Notably, all 3 cases were complicated by preexisting lung conditions, such as asthma in case-patient 1, or a co-existing infection, such as influenza A(H1N1) in case-patient 2 and group A streptococcus in case-patient 3. Although the severity of these cases may have been influenced by these concomitant conditions, many virus infections are known to have a more severe course during pregnancy. Perhaps the most compelling evidence to suggest that RSV might be more virulent during pregnancy is that infections with many viruses (e.g., varicella-zoster, hepatitis, and most notably, influenza) are known to have a more severe course in pregnant women (*10**–**12*). The prevalence of influenza in pregnancy is similar to that in the general population, yet influenza carries a 5-fold increased risk of death in pregnant women (*13*). The pathophysiology underlying the increased risk for illness and death caused by influenza during pregnancy remains poorly understood. The immunologic changes that allow a genetically distinct fetus to inhabit a mother with a functioning immune system likely contribute greatly to the increased illness and death caused by influenza in pregnancy. Cytotoxic adaptive immunity, which is involved in defense against virus infection, is diminished during pregnancy, whereas the regulatory adaptive immune system, the basis for immune memory and vaccine immunology, is heightened (*14**–**17*).

Despite the potential for devastating consequences, RSV infection in pregnant women has likely been unrecognized, as this infection once was in the elderly population. Falsey et al. (*1*) reported RSV infection in 4% to 10% of high risk adults annually, among whom >85% were symptomatic, 16%% required hospitalization, and 4% died. They also estimated that the disease prevalence of RSV infection in immunocompromised and elderly adults is similar to that of nonpandemic influenza (*1*). 

A major barrier to the effective diagnosis of RSV is the inability to make the diagnosis solely on the basis of clinical features, which are similar to those of infections caused by adenovirus, rhinovirus, parainfluenza virus, or influenza virus. The reference standard for diagnosis (i.e., isolation of the virus in cell culture and identification of plaque morphologic features with syncytium formation) is time consuming and expensive. More recently, PCR-based testing has become more available and allows for a more rapid and specific diagnosis. Laboratory testing has only recently been adopted in academic hospital centers and is still underused in the community setting. Although RSV subtype A typically causes more severe disease than RSV subtype B (*18*), the PCR-based test used in our institution does not allow for RSV subtyping. As was evident from the cases presented, co-infection with other viruses or bacteria can also add to diagnostic challenges and severity of clinical features.

Although the cases presented occurred at a single institution during a single RSV season, no overall increase in RSV incidence or severity occurred among the general population of children and adults that would explain an increase in incidence among the pregnant population. With the increased availability of viral PCR panels that detect RSV, we suspect that we are now recognizing an infection that has long been present. Once RSV infection is suspected and diagnosed in pregnancy, treatment is also more complicated. Although the cornerstone of RSV treatment is supportive therapy, disease management can include administration of ribavirin, a nucleoside analog that has been shown to improve outcomes in severely immunocompromised RSV-infected adults (*19*). However, ribavirin is contraindicated in pregnancy because of its teratogenic effects, which have been documented in multiple animal studies up to 7 months after cessation of treatment (*20*), but these findings are unsupported by human data. The Ribavirin Pregnancy Registry reported 6 cases of birth defects in 49 live births (over a 5-year surveillance period) in which the mother received ribavirin. The birth defects included polydactyly, glucose 6-phosphate dehydrogenase deficiency, hypospadias, ventricular septal defect, cyst of the fourth ventricle of the brain, and torticollis (*21*). Although the evidence remains limited, ribavirin is currently classified by the US Food and Drug Administration as pregnancy category X drug. Nonetheless, despite safety concerns, in the setting of maternal life-threatening illness, antiviral agents should be considered, given that fetal well-being is ultimately maximized by maternal well-being.

No data have documented vertical RSV transmission in humans, and in other viral illnesses, such as influenza, this phenomenon is rare (*22**,**23*). Fortunately, none of the infants of the case-patients described herein had any signs of RSV infection.

Vaccine trials are currently underway, and questions like the following will inevitably arise: Who should get the vaccine? At what gestational age should it be administered? Is the vaccine cost effective? For pregnant women, RSV infection may pose a substantial risk for hospitalization and further complications, and the infection is likely worsened in the setting of baseline pulmonary disease, such as asthma, and tobacco use (*24*). The cases presented here demonstrate the wide spectrum of maternal disease and emphasize the potential for maternal benefit of an RSV vaccine administered during pregnancy. However, studies that can characterize the maternal effects of RSV infection at the population-level are necessary to answer these questions and enable any effective RSV vaccine to be used to its full potential to protect mother and infant.
